# Assembly processes and network structure of rhizosphere fungal communities in healthy and *Ralstonia solanacearum*-infected tomato plants

**DOI:** 10.3389/fpls.2026.1956668

**Published:** 2026-10-05

**Authors:** Caixia Liu, Mengli Zhao, Qingying Gao, Chunju Peng, Liang Wang, Jie Yang

**Affiliations:** 1 Instrumental Analysis Center, Wenzhou Academy of Agricultural Sciences, Wenzhou, China; 2 Key Laboratory of Soil Remediation and Quality Improvement of Zhejiang Province, National Key Laboratory for Development and Utilization of Forest Food Resources, College of Environment and Resources/College of Carbon Neutrality, Zhejiang A&F University, Hangzhou, China; 3 Department of Landscape and Hydraulic Engineering, Wenzhou Academy of Agricultural Sciences, Wenzhou, China; 4 Institute of Bamboo Research, Fujian Academy of Forestry Sciences, Fuzhou, China

**Keywords:** community assembly, co-occurrence network, fungal community, plant disease, plant rhizosphere

## Abstract

**Introduction:**

Bacterial wilt caused by *Ralstonia solanacearum* is a major constraint to tomato production worldwide. Rhizosphere microbial community is closely linked to plant health. However, how *R. solanacearum* invasion alters the assembly processes and co-occurrence network in tomato rhizosphere fungal communities remains largely unclear.

**Methods:**

Here, we collected rhizosphere soil samples from three major tomato-producing regions in China to investigate how *R. solanacearum* invasion affects fungal community composition, network topology, and assembly processes in tomato rhizosphere.

**Results and discussion:**

In this study, fungal community structure differed significantly between healthy and diseased rhizosphere soils, driven significantly by soil pH (*P* < 0.05). The density of pathogen *R. solanacearum* in the diseased rhizosphere soil was significantly higher than that in the healthy tomato rhizosphere (*P* < 0.05). Genera *Trichoderma*, *Talaromyces*, *Mortierella*, and *Penicillium* were significantly enriched in healthy soils (*P* < 0.05). Network analysis revealed diseased soils had simpler and less stable fungal networks, with decreased node and edge numbers, reduced natural connectivity, and a smaller proportion of connectors. The neutral community model and null model revealed that stochastic processes explained 33.10% of the community variation in healthy tomato rhizosphere fungal communities, whereas they explained 47.40% in diseased soils, with a higher migration rate (*m*) in diseased soils (0.0050) than in healthy soils (0.0027). These results indicate that fungal assembly was governed by deterministic processes in healthy soils, while stochastic processes dominated in diseased soils. The fungal communities in diseased soils exhibited narrower niche breadth, and lower proportions of generalists and specialists than those in healthy soils. Collectively, these findings indicate that *R. solanacearum* invasion leads to niche contraction, reduced generalists and specialists, and increased migration rates in fungal communities. These changes render species coexistence more dependent on dispersal and drift, shifting community assembly from deterministic to stochastic processes and potentially reducing network stability. This study provides deeper insights into soil fungal community assembly and structure following *R. solanacearum* infection in tomato.

## 1 Introduction

Plants recruit specific beneficial microorganisms by releasing root exudates to enhance nutrient acquisition, improve tolerance to abiotic stresses, and prevent diseases by inducing immune responses to antagonize pathogens (Manathunga et al., 2024; Liu et al., 2026). The rhizosphere microbiome is considered the plant’s “second genome,” with its composition and functional expression closely tied to plant health (Manathunga et al., 2024). However, pathogens do not invade plant rhizospheres alone but synergize with opportunistic microbes to form a “pathobiome”, accelerating disease development (Arnault et al., 2023; Ai et al., 2026). Inspired by Leo Tolstoy’s dictum, the Anna Karenina principle has been applied to plant microbiota: “All healthy microbiota are alike; each disease-associated microbiota is diseased in its own way,” implying that microbial communities in diseased individuals may exhibit greater inter-individual variation (Li et al., 2026). Therefore, elucidating the differences in community structure, networks, and assembly processes between healthy and diseased rhizosphere microbiomes is essential for understanding soil-borne disease dynamics and developing effective biological control strategies.

Unraveling the assembly mechanisms of microbial communities is essential for explaining their formation, succession, and the maintenance of ecosystem functions (Santillan et al., 2025). Determining the relative contributions of deterministic (e.g., host filtering, environmental filtering, competition) and stochastic processes (e.g., drift, dispersal limitation) to community assembly is a key question in microbial ecology (Jiao et al., 2020). When resources are plentiful and environmental conditions are stable, reduced microbial competition may favor stochastic processes. Conversely, when environmental heterogeneity increases or resources are limited, intensified microbial competition strengthens deterministic selection (Liu et al., 2023). The neutral community model (NCM) and C-score analysis based on null models are commonly applied to quantify the relative roles of deterministic and stochastic processes in microbial community assembly. A higher *R²* of the NCM indicates stronger stochasticity, while a higher standardized effect size (SES) of the C-score (|SES| > 2) indicates deterministic dominance (Mo et al., 2021). Pathogen invasion of the rhizosphere perturbs the micro-environment and host filtering conditions, consequently redirecting the assembly processes of soil microbial community (Wen et al., 2022; Ai et al., 2026). Elucidating how pathogen invasion alters the relative contributions of deterministic and stochastic processes to rhizosphere microbial community assembly is critical for understanding shifts in community composition and functional decline (Ai et al., 2026). Microbial co-occurrence network analysis provides a quantitative tool for elucidating potential microbial interactions (e.g., mutualism, competition, and antagonism) across diverse complex environments, including marine ecosystems, E-waste dismantling areas, and animal guts (Ruan et al., 2006; Hao et al., 2025). Network topological features (e.g., node count, edge count, natural connectivity, and connector proportion) directly indicate community complexity and resilience (Jiao et al., 2020; Zhang et al., 2025). Furthermore, investigating the coexistence strategies of taxa within microbial communities contributes to reveal the ecological assembly processes of these communities (Zhang et al., 2020). Integrating community assembly analysis with network approaches provides insights into microbial community structure formation following pathogen invasion from two perspectives: the deterministic–stochastic trade-off and species interactions (Chen et al., 2024; Han et al., 2025). Fungi are an important member of the soil microbiome, typically accounting for over 40% of its microbial biomass (Tedersoo et al., 2020). Nevertheless, most rhizosphere microbiome studies have predominantly concentrated on bacterial communities (Han et al., 2025). The assembly mechanisms of structurally complex fungal communities and their associations with network topology during disease progression remain largely unexplored.

Tomato (*Solanum lycopersicum*) is among the most widely grown vegetable crops globally. Bacterial wilt caused by *Ralstonia solanacearum* leads to substantial yield and economic losses in tomato production (Tans-Kersten et al., 2001; Duan et al., 2026). Some studies have focused on the pathogenic mechanisms of *R. solanacearum* and the screening of antagonistic strains (Zhao et al., 2019; Luo et al., 2026). However, how pathogen invasion alters the community structure, assembly processes, network topology, and their interconnections in tomato rhizosphere fungal communities remains poorly understood. In this study, rhizosphere soils were sampled from *R. solanacearum*-infected and healthy tomato plants across three major tomato-producing regions in China. Fungal community composition, assembly processes, and co-occurrence networks were compared between healthy and *R. solanacearum*-infected tomato rhizosphere soils by integrating ecological niche theory, NCM, and network analysis. We proposed two hypotheses: (1) healthy and diseased tomato rhizosphere soils differed markedly in fungal community structure, assembly, and network topology; and (2) deterministic selection led to more complex and connected fungal co-occurrence networks, whereas stochastic processes resulted in simpler ones. fungal community assembly processes may be closely associated with network structure. This study aims to clarify the variation and response of rhizosphere fungal communities to *R. solanacearum* invasion, providing a theoretical basis for the targeted regulation of rhizosphere fungal communities.

## 2 Materials and methods

### 2.1 Experimental design and sampling

Rhizosphere soil samples from both healthy and diseased tomato plants (infected with *R. solanacearum*) were collected from Cangnan County (27°30′N, 120°23′E), Longgang City (27°30′N, 120°23’E), and Rui’an City (27°40′N, 120°10′E) in Zhejiang Province, China (**Supplementary Figure S1**). Three sampling regions are well-known for greenhouse tomato production in China. The three regions are characterized by a subtropical maritime monsoon climate, with mean annual temperatures of 18.0 °C, 17.9°C and 17.9 °C, and annual precipitation of 1753.9 mm, 1670.1, mm and 1753.9 mm, respectively. According to the World Reference Base (WRB) classification, the soils in the study region are identified as Gleyic Fluvisols, which have been converted from paddy soils. These soils typically have a silty clay loam texture. Detailed geographical and fertilization information for the sampling regions is shown in **Supplementary Table S1**. All sampled greenhouses followed standard cultivation practices, including tillage, fertilization, and pesticide application. For basal fertilization, each greenhouse utilized a combined application of organic and chemical fertilizers for tomato cultivation. The organic amendments comprised chicken manure, sheep manure, and commercial organic fertilizers. The chicken and sheep manure, procured from commercial animal farms, underwent a standardized four-week open-air composting process prior to application. The nutrient contents of chicken manure were on average 340 g kg^−1^ of organic C, 28.30 g kg^−1^ of total N, 5.48 g kg^−1^ of P_2_O_5_ and 3.96 g kg^−1^ of K_2_O. The nutrients of sheep manure were on average 450 g kg^−1^ of organic C, 16.50 g kg^−1^ of total N, 4.52 g kg^−1^ of P_2_O_5_ and 1.62 g kg^−1^ of K_2_O. The nutrients contents of commercial organic fertilizer were on average 370 g kg^−1^ of organic C, 2.01 g kg^−1^ of total N, 2.31 g kg^−1^ of total P and 1.21 g kg^−1^ of total K. This fertilizer was commercially sourced from an organic fertilizer manufacturer, with its primary composition comprising agricultural wastes (i.e. cattle manure, chicken manure, cotton hulls, and crop straws), which underwent microbial fermentation and controlled decomposition processes.

Rhizosphere soil samples were obtained in May 2024. Ten greenhouses with 10 – 13 years of tomato planting history were selected in each of the three sampling sites (Rui’an, Cangnan, and Longgang), totaling 30 greenhouses. In each greenhouse, the roots of five diseased plants and five healthy plants were carefully excavated and pooled separately to form one diseased sample and one healthy sample, respectively. Diseased plants were selected based on typical bacterial wilt symptoms, including uppermost leaves wilting and drooping while remaining green, and brown vascular bundles. Healthy plants grew normally with no leaf wilting. Sampling tools were not shared between individual plants. Disposable gloves were changed between plants. A physical distance of at least 2 m was maintained between the sampling positions of healthy and diseased plant. A total of 60 rhizosphere samples (30 from diseased plants and 30 from healthy plants) were collected from 30 greenhouses. Then, adhered soil around the root was loosely removed by shaking. All samples were placed in sterile bags, stored in a cooler, and transported to the laboratory at 4 °C. The collected tomato roots were cut into approximately 2 cm segments. Subsequently, the root segments were transferred to a triangular flask containing 200 mL of sterilized water. The triangular flask was shaken (170 rpm, 30 °C, 30 min), followed by 100 Hz ultrasonic treatment for 10 min. After removing the roots with sterilized tweezers, the soil suspension was centrifuged at 10,000 rpm at 4 °C for 10 min. The collected precipitate was stored at −80 °C for microbial genomic DNA extraction.

### 2.2 Analysis of soil physicochemical properties and enzyme activities

Soil microbial biomass carbon (MBC) and nitrogen (MBN) were determined using the chloroform fumigation-extraction method (Vance et al., 1987; Brookes et al., 1985). The measurement methods for soil pH, soil organic carbon (SOC), total nitrogen (TN), alkaline hydrolyzable nitrogen (AN), available phosphorus (AP), and available potassium (AK) followed the protocols described in Bao (2000). Dissolved organic carbon (DOC) was extracted with 0.5 mol L^-1^ K_2_SO_4_ at a soil to solution ratio of 1:4 (w/v), and the filtered solution was measured using a total organic carbon (TOC) analyzer (Shimadzu TOC-L CPH, Japan). Activities of four carbon-degrading enzymes (α-glucosidase, AG; β-glucosidase, BG; β-D-cellobiosidase, CB; xylanase, XYL), two nitrogen-degrading enzymes (β-N-acetyl glucosaminidase, NAG; leucine aminopeptidase, LAP), and one phosphorus-degrading enzyme (acid phosphatase, PHOS) were assayed following Saiya-Cork et al. (2002). Enzyme activities were measured fluorometrically with substrates labeled with 4-methylumbelliferone (MUB) and 7-amino-4-methylcoumarin (MUC). Enzyme activities were expressed in nmol g^−1^ dry soil h^−1^ (Chen et al., 2019). The geometric mean of enzyme activity (GMEA) was calculated based on the assayed enzyme activities for each sample to give total enzyme activity.

### 2.3 The density of *R. solanacearum* in the rhizosphere soils of both healthy and diseased tomatoes

The density of *R. solanacearum* was measured by semi-selective medium South Africa (SMSA) medium, which was specifically designed for the isolation of *R. solanacearum* from complex substrates such as soil (Elphinstone et al., 1996). SMSA medium was supplemented with multiple antibiotics and dyes, including TZC (2,3,5-triphenyl tetrazolium chloride), crystal violet, polymyxin, cycloheximide, penicillin, chloramphenicol, and bacitracin, which effectively inhibit the growth of Gram-positive bacteria, some Gram-negative bacteria, and fungi, thereby allowing *R. solanacearum* to become the dominant colonies on the plates. TZC (2,3,5-triphenyl tetrazolium chloride) was added to the SMSA medium at a final concentration of 50 mg L^-1^, serving as a redox indicator that is reduced by *R. solanacearum*, producing a pink to blood-red coloration that further aids in distinguishing colony types. The dilution plating procedure followed the protocol of Elphinstone et al. (1996). Ten grams tomato roots were added to 90 mL sterile water, and a 10^-4^ to 10^-6^ soil suspension was prepared by successive tenfold dilutions. Each dilution was plated in triplicate, and 100 μL of the suspension was spread onto the plates, then they were incubated in darkness at 28 °C for 3 days. After incubation, typical colonies of virulent *R. solanacearum* appeared, characterized by irregular, rounded, and fluidal morphology with a pink center (Wei et al., 2013) (**Figure 1A**). Using the semi-selective South Africa medium (SMSA), *R. solanacearum* colonies can be distinguished based on colony morphology and color (Wei et al., 2013). The colony-forming units (CFUs) of *R. solanacearum* were identified and recorded.

**Figure 1:**
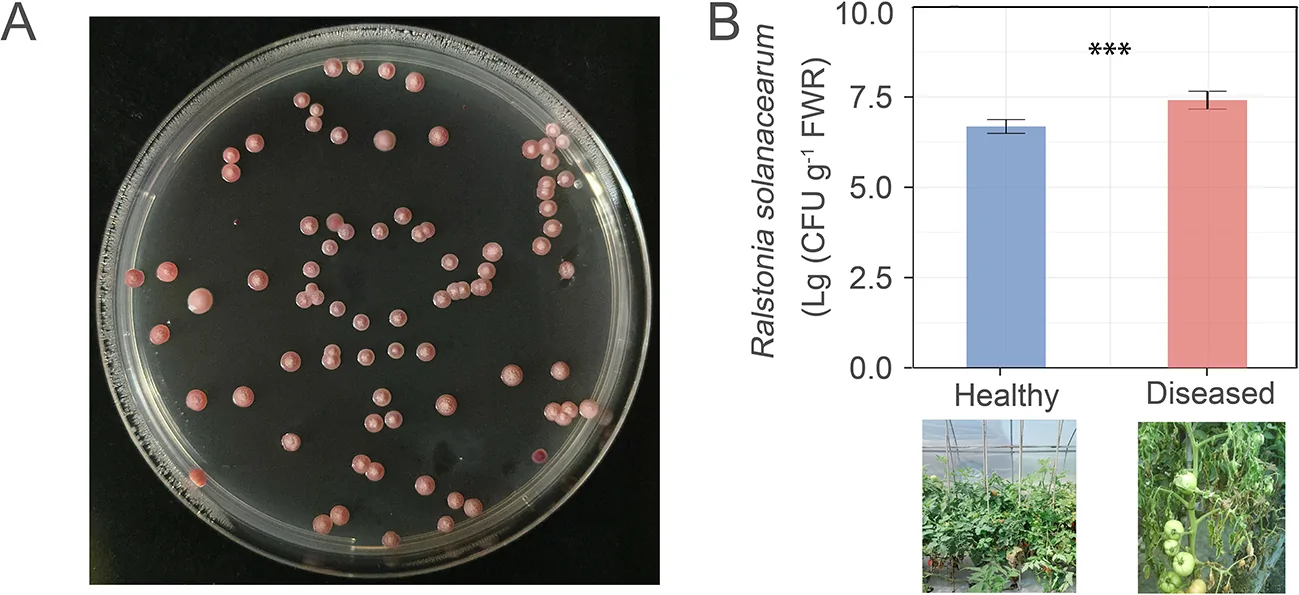
*R. solanacearum* density and fungal community structure in healthy and diseased tomato rhizosphere soils. **(A)** Morphology of *R. solanacearum* colonies on semi-selective medium South Africa (SMSA) medium. **(B)** *R. solanacearum* density. *P < 0.05; **P < 0.01; ***P < 0.001. n = 30.

### 2.4 DNA extraction and sequencing

Total genomic DNA was extracted and assessed for quality as described by Liu et al. (2023). High-throughput sequencing targeting the fungal ITS1 region was performed using primers ITS1 (5’-TCCGTAGGTGAACCTGCGG-3’) and ITS2 (5’-GCTGCGTTCTTCATCGATGC-3’) to assess fungal diversity and community composition (Gardes and Bruns, 1993). The 25 μL PCR reaction was performed by 12.5 μL of 2× Buffer Mix (contains DNA Polymerase, dNTPs and buffer), 1 μL of template DNA (10 ng), 1 μL of each primer (10 μM), and 9.5 μL of ddH2O. All amplified PCR products were examined by agarose gel electrophoresis (1.5%), purified, and subjected to paired-end sequencing on the Illumina PE300 platform (Wekemo Tech Group Co., Ltd., Shenzhen, China).

Sequence processing was performed using QIIME (v.1.9.0). Sequences with a length < 50 bp, average quality score < 30, or mismatches in the barcode/primer regions were removed. Paired-end reads were assembled with FLASH (Fast Length Adjustment of Short) reads, requiring at least 10 bp of overlap and ≤5% mismatches in the overlapping region. Chloroplast and mitochondrial sequences were removed, and the remaining high-quality reads were clustered into amplicon sequence variants (ASVs) at 99% sequence similarity. Taxonomic assignment was performed by BLASTn against the UNITE eukaryotic database (https://unite.ut.ee/). To rigorously exclude non-fungal sequences, only ASVs assigned to the Kingdom Fungi were retained for downstream analyses, while sequences classified as non-fungal or unassigned at the kingdom level were discarded. Raw sequencing data have been deposited in the NCBI Sequence Read Archive (BioProject accession: PRJNA1463964).

### 2.5 Statistical analysis

The NCM was fitted to ASV occurrence frequency and relative abundance data to quantify stochastic contributions to fungal community assembly (Sloan et al., 2006). Model fit was evaluated using *R²*, calculated following Ostman et al. (2010), with 95% confidence intervals derived from 1000 bootstrap replicates. All computations were performed in R (version 3.5.3). The parameter *Nm* represents the dispersal rate among communities. The parameter *N* estimates the metacommunity size. The migration rate (*m*) is the probability of an ASV being replaced by metacommunity dispersal following local extinction. Higher *m* values indicate weaker dispersal limitation.

To examine fungal co-occurrence patterns, we employed a null model framework based on the C-score (Stone and Roberts, 1990). For healthy and diseased tomato rhizosphere soil samples, we constructed presence–absence matrices from the ASV abundance tables (samples as rows, ASVs as columns), which were then used as input for the null model. Null matrices were generated using the proportional row–proportional columns algorithm implemented in the “EcoSimR” R package (version 4.0.5). The SES, obtained from the null model, was calculated to assess the relative contribution of deterministic processes to community assembly (Mo et al., 2021). SES > 2 indicates overdispersion (aggregation), while SES < −2 indicates underdispersion (segregation), reflecting the relative importance of deterministic processes (Mo et al., 2021). |SES| ≤ 2 indicates that the observed co-occurrence pattern does not deviate significantly from the null expectation.

Levin’s niche breadth (*B*) index was calculated according to the following formula:


$$B_{j}=1/\sum_{i=1}^{N}P_{ij}^{2}$$

where *p_ij_* is the relative abundance of ASV *j* in community *i*, and *N* is the total number of communities. Higher B values indicate broader and more even habitat distribution and niche breadth (Feinsinger et al., 1981). Community-level niche breadth (*Bcom*) was determined by averaging *B* values of all ASVs per community with the “spaa” R package (version 3.5.3) (Mo et al., 2021). ASVs were classified into generalists and specialists via 1000 permutations (null distributions) using “EcolUtils” R package (version 3.5.3). ASVs were classified as generalists (observed frequency > upper 95% confidence interval), specialists (observed frequency < lower 95% confidence interval), or neutral (within 95% confidence interval) based on null distributions (Xu et al., 2022).

Co-occurrence networks were constructed with a correlation coefficient |*r*| > 0.60 and a false discovery rate (FDR)-adjusted *P* value < 0.05 Using the WGCNA package (version 3.5.3) (Jiao et al., 2020). Fungal ASVs with a mean relative abundance exceeding 0.5% were selected for network construction. Topological features, including the number of nodes, number of edges, average degree, clustering coefficient, betweenness centrality, and modularity, natural connectivity were quantified using the “igraph” package in R (version 3.5.3) (Jun et al., 2010). Nodes indicate microbial taxa, and edges represent significant correlations between them. Average degree indicates the overall connectivity of the network. Natural connectivity evaluated the overall robustness and resilience of the network to node removal, with higher values indicating greater stability. Networks were visualized using Gephi (version 0.9.2) (http://gephi.github.io/). Keystone taxa were identified by calculating two topological parameters for each node: within-module connectivity (Zi) and among-module connectivity (Pi), which reflect a node’s linkage to other nodes within its own module and to nodes in other modules, respectively. Based on Zi and Pi thresholds, nodes were assigned to one of four categories: network hubs (Zi > 2.5, Pi > 0.62), module hubs (Zi > 2.5, Pi ≤ 0.62), connectors (Zi ≤ 2.5, Pi > 0.62), and peripherals (Zi ≤ 2.5, Pi ≤ 0.62) (Deng et al., 2012). Network hubs, module hubs, and connectors were grouped as keystone taxa due to their critical roles in sustaining network structure and community function (Deng et al., 2012).

One-way analysis of variance (ANOVA) was used to examine soil properties, microbial biomass, enzyme activities, and fungal diversity using SPSS 20.0 (SPSS Inc., Chicago, IL, USA). The differences in the fungal community composition were assessed by principal coordinate analysis (PCoA) based on community Bray-Curtis distances and permutational multivariate analysis (PERMANOVA) using the “vegan” R package (version 3.5.3). Linear discriminant analysis Effect Size (LEfSe) was applied to detect fungal taxa showing significant abundance differences between healthy and diseased tomato rhizosphere soils. Taxa with linear discriminant analysis (LDA) scores > 2 and *P* < 0.05 were considered statistically significant biomarkers (Segata et al., 2011). The associations between fungal community structure and soil physicochemical properties, microbial biomass, and enzyme activities were evaluated by Mantel tests, which were based on Euclidean distance matrices for environmental variables and Bray-Curtis dissimilarity matrices for fungal community composition. Pearson’s correlation calculated for soil physicochemical properties, microbial biomass, and enzyme activities. The combined results of Pearson’s correlation and Mantel tests were visualized using the “ggcor” package in R (version 4.0.5).

## 3 Results

### 3.1 Soil physicochemical properties, microbial biomass and enzyme activities between healthy and diseased rhizosphere soils

The contents of SOC and AN were significantly higher in healthy rhizosphere soils than in diseased soils (*P* < 0.05), whereas pH, TN, AP, AK, and DOC showed no significant differences between the two groups. MBC and MBN were significantly higher in diseased rhizosphere soils than in healthy soils (*P* < 0.05). The activities of AG and LAP were significantly higher in healthy soils than in diseased soils (*P* < 0.05) (**Table 1**).

**Table 1:** Soil physicochemical properties, microbial biomass, and enzyme activities in healthy and diseased tomato rhizosphere soils.

Soil physicochemical properties	Healthy	Diseased	*F*	*P*
pH (H_2_O)	5.94 ± 0.90	6.11 ± 0.78	0.59	0.45
TN (g kg^-1^)	3.12 ± 1.65	2.51 ± 0.57	3.65	0.06
SOC (g kg^-1^)	58.39 ± 28.60	46.39 ± 8.03	4.90	0.03
AN (mg kg^-1^)	192.78 ± 68.48	163.49 ± 33.29	4.44	0.04
AP (mg kg^-1^)	266.09 ± 105.02	294.98 ± 156.82	0.70	0.41
AK (mg kg^-1^)	749.13 ± 588.38	788.77 ± 393.66	0.09	0.76
DOC (mg kg^-1^)	987.45 ± 560.90	1042.5 ± 441.83	0.18	0.67
MBC (mg kg^-1^)	345.84 ± 198.13	590.62 ± 212.94	21.25	0.00
MBN (mg kg^-1^)	73.6 ± 44.36	195.78 ± 205.11	10.17	0.00
AG (nmol g^-1^dry soil h^-1^)	1.77 ± 1.10	2.39 ± 1.22	4.33	0.04
BG (nmol g^-1^dry soil h^-1^)	52.22 ± 24.85	50.7 ± 21.58	0.06	0.80
CB (nmol g^-1^dry soil h^-1^)	2.52 ± 1.66	3.12 ± 2.01	1.60	0.21
XYL (nmol g^-1^dry soil h^-1^)	5.10 ± 2.90	5.01 ± 2.88	0.02	0.90
NGA (nmol g^-1^dry soil h^-1^)	37.65 ± 12.44	36.15 ± 14.02	0.19	0.66
LAP (nmol g^-1^dry soil h^-1^)	5.73 ± 2.14	7.54 ± 1.53	14.15	0.00
PHOS (nmol g^-1^dry soil h^-1^)	163.36 ± 65.15	166.74 ± 29.38	0.07	0.80
GMEA	11.56 ± 4.74	13.25 ± 5.07	1.78	0.19

Mean ± standard deviation, n = 30. SOC, soil organic carbon; TN, total nitrogen; AN, Alkaline hydrolyzable nitrogen; AP, available phosphorus; AK, available potassium; DOC, dissolved organic carbon; MBC, microbial biomass carbon; MBN, microbial biomass nitrogen. AG, α-glucosidase; BG, β-glucosidase; CB, β-D-cellobiosidase; XYL, β-xylosidase; NAG, N-acetyl-β-glucosaminidase; LAP, leucine aminopeptidase; PHOS, phosphatase; GEMA, geometric mean enzyme activity. .

### 3.2 *R. solanacearum* density and fungal community structure in healthy and diseased tomato rhizosphere soils

The density of pathogen *R. solanacearum* in the diseased rhizosphere soil was significantly higher than that in the healthy tomato rhizosphere (*P* < 0.05) (**Figure 1B**). A total of 5,788,099 high-quality fungal reads were obtained across 60 samples, with per-sample read counts ranging from 52,108 to 141,799 (mean = 94,887). These reads were clustered into 2,045 ASVs. The Shannon diversity index of fungal communities was significantly higher in healthy tomato rhizosphere soils than in diseased soils (*P* < 0.05) (**Figure 2A**). Fungal community structure differed significantly between healthy and diseased tomato rhizosphere soils (**Figure 2B**). At the phylum level, the fungal communities were dominated by Ascomycota (29.36%) and Basidiomycota (2.41%). The relative abundance of Ascomycota and Basidiomycota was higher in healthy soils (36.70% and 3.32%) than in diseased soils (22.02% and 1.50%) (**Figure 2C**). LEfSe analysis identified differentially enriched taxa between the two groups (**Figure 2D**). In healthy rhizosphere soils, significantly enriched taxa included *Penicillium* (Genus), *Arthrobotrys* (Genus), Chaetothyriales (Order), *Rhodotorula* (Genus), Sporidiobolaceae (Family), Sporidiobolales (Order), Microbotryomycetes (Class), Helotiales (Order), Leotiomycetes (Class), *Scytalidium* (Genus), Helotiaceae (Family), Zoopagales (Order), Zoopagomycota (Phylum), *Syncephalis* (Genus), Zoopagomycetes (Class), and Piptocephalidaceae (Family) (LDA > 2, *P* < 0.05). In diseased rhizosphere soils, significantly enriched taxa included *Helicoma* (Genus), Tubeufiaceae (Family), Tubeufiales (Order), Trichosporonales (Order), Trichosporonaceae (Family), *Macrophomina* (Genus), Aureobasidiaceae (Family), Dothideales (Order), *Aureobasidium* (Genus), and Dothideomycetes (Class) (LDA > 2, *P* < 0.05) (**Figure 2D**).

**Figure 2:**
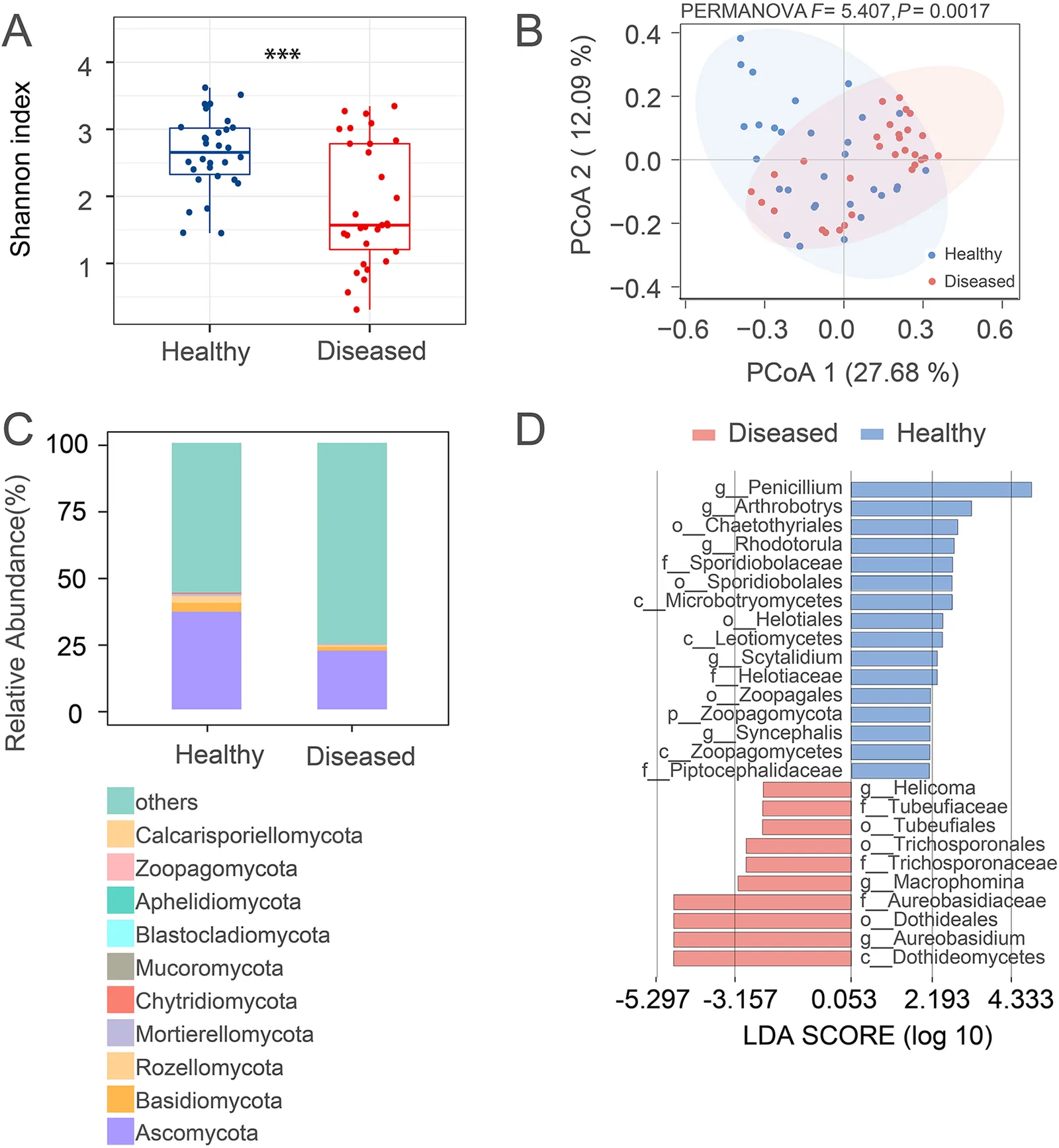
*R. solanacearum* density and fungal community structure in healthy and diseased tomato rhizosphere soils. **(A)** Fungal Shannon index. **(B)** Principal coordinate analysis (PCoA) based on Bray-Curtis distances showing fungal community structure differences between healthy and diseased tomato rhizosphere soils. **(C)** Relative abundance of fungal phyla. **(D)** Histogram of linear discriminant analysis (LDA) scores (log10) for fungal taxa with significant differential abundance between the two groups (LDA > 2.0, *P* < 0.05). Blue bars represent taxa enriched in healthy soils; red bars represent taxa enriched in diseased soils. *P < 0.05; **P < 0.01; ***P < 0.001. n = 30.

Mantel tests showed that soil pH, NAG, LAP, and GEMA were significantly correlated with fungal community variation in healthy tomato rhizosphere soils (*P* < 0.05), while only soil pH was significantly correlated with fungal community variation in diseased tomato rhizosphere soils (*P* < 0.05) (**Figure 3**). Pearson’s correlation analysis revealed that MBC was positively correlated with AK (*P* < 0.05), whereas MBN showed no significant correlation with any of the measured variables. AG had a positive and significant correlation with TN, SOC, AN, DOC and MBC (*P* < 0.05). BG was positively and significantly correlated with TN, AN and AK (*P* < 0.05). CB and XYL exerted a positive and significant correlation with AN (*P* < 0.05). NAG was positively and significantly correlated with TN, SOC, AN and AK (*P* < 0.05). LAP was negatively and significantly correlated with TN, SOC, AN, pH and AK (*P* < 0.05). PHOS had a negative and significant correlation with pH and DOC (*P* < 0.05).

**Figure 3:**
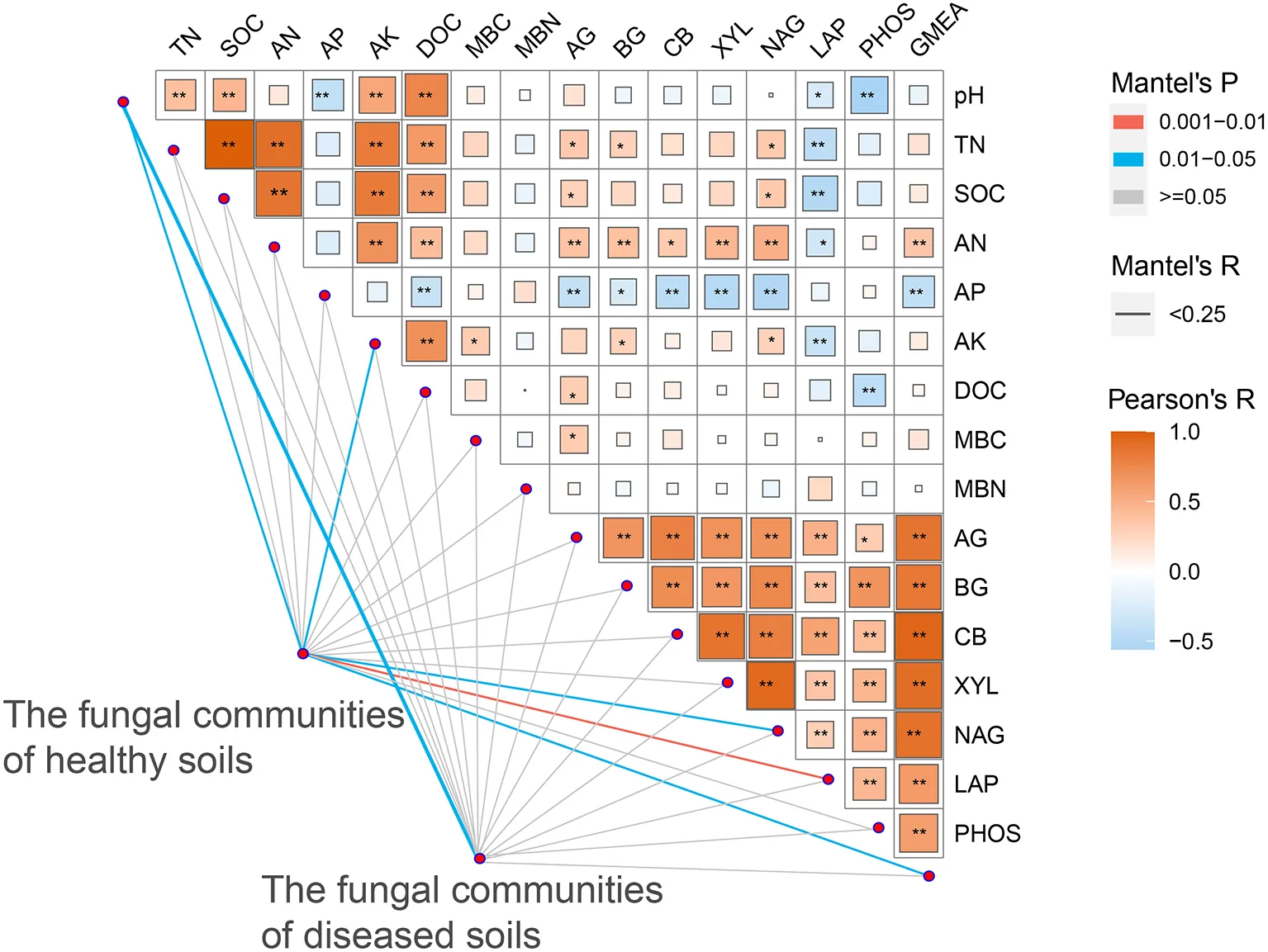
Relationships between soil physicochemical properties, microbial biomass, enzyme activities, and fungal community structure in healthy and diseased tomato rhizosphere soils. Mantel test showing correlations between fungal community structure (based on Bray-Curtis dissimilarity) and soil variables, including physicochemical properties, microbial biomass, and enzyme activities. Spearman correlation coefficients between soil variables are represented by color gradients and square size. SOC, soil organic carbon; TN, total nitrogen; AN, alkaline hydrolyzable nitrogen; AP, available phosphorus; AK, available potassium; DOC, dissolved organic carbon; MBC, microbial biomass carbon; MBN, microbial biomass nitrogen. AG, α-glucosidase; BG, β-glucosidase; CB, β-D-cellobiosidase; XYL, β-xylosidase; NAG, N-acetyl-β-glucosaminidase; LAP, leucine aminopeptidase; PHOS, phosphatase; GMEA, geometric mean enzyme activity. *P < 0.05, **P < 0.01, ***P < 0.001.

### 3.3 The co-occurrence patterns of fungal community in the healthy and the diseased rhizosphere soils

The co-occurrence patterns of fungal communities were constructed from ASV correlations in healthy and diseased soils (**Figure 4A**; **Supplementary Table S2**). The co-occurrence network pattern of fungi in healthy tomato rhizosphere soils differed profoundly from that in diseased soils. The healthy networks showed higher node and edge numbers, as well as enhanced natural connectivity than diseased networks (**Figures 4B**, **2C**). A total of 50 and 18 connectors were identified in the fungal co-occurrence networks of healthy and diseased tomato rhizosphere soils, representing 50.63% and 29.03% of the total nodes, respectively (**Figure 4D**; **Supplementary Tables S3, S4**). These connector taxa were predominantly affiliated with Ascomycota, Basidiomycota, and Mortierellomycota (**Supplementary Tables S3, S4**).

**Figure 4:**
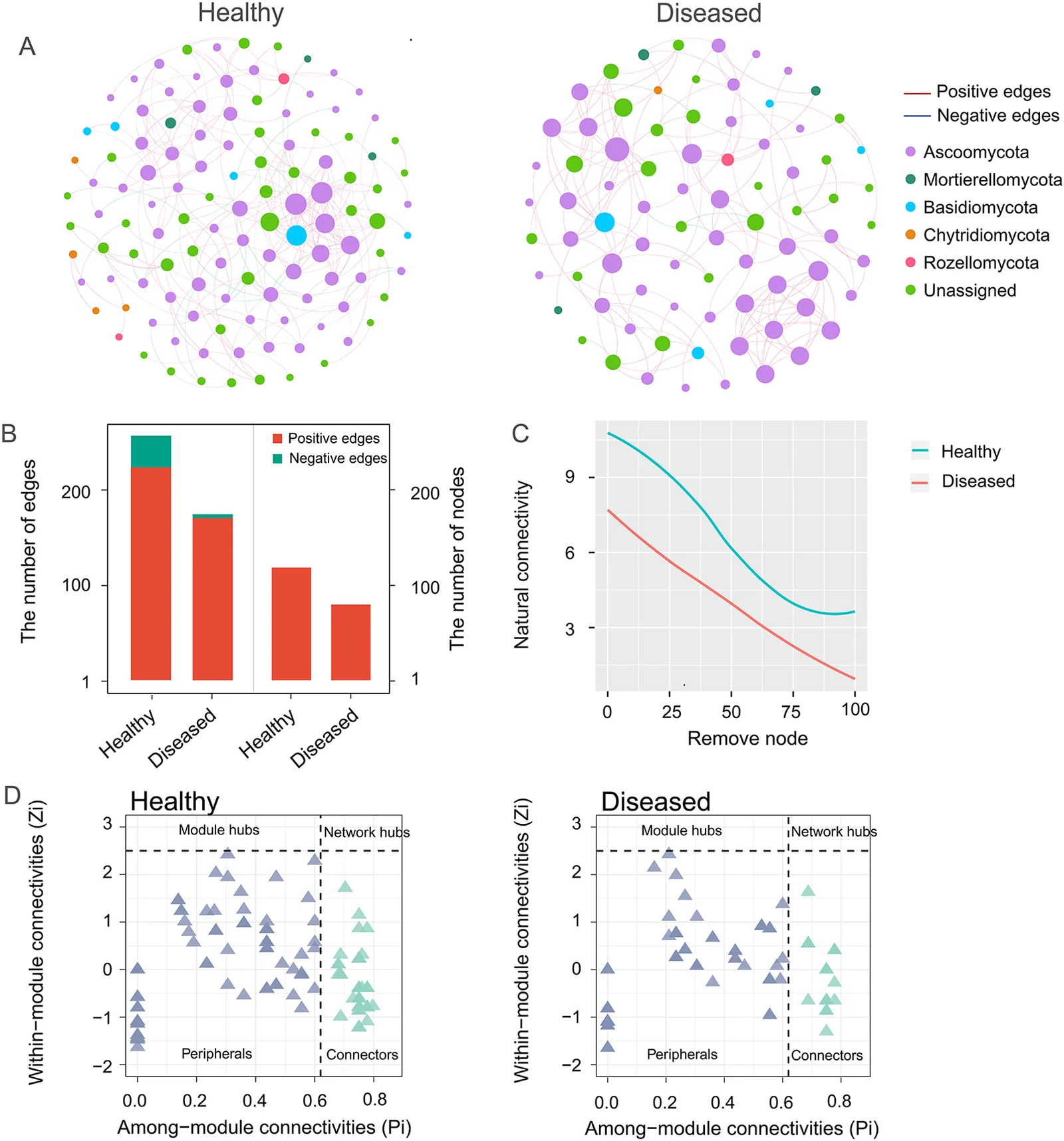
Co-occurrence network patterns **(A)**, edge and node properties **(B)**, natural connectivity **(C)**, and keystone taxa distribution **(D)** of fungal communities in healthy and diseased tomato rhizosphere soils.

### 3.4 Community assembly processes of fungal communities in healthy and diseased tomato rhizosphere soils

The NCM revealed that stochastic processes explained 33.10% of the community variation in healthy tomato rhizosphere fungal communities, whereas they explained 47.40% in diseased tomato rhizosphere fungal communities (**Figure 4A**). The *m* value in diseased rhizosphere soils was 1.85 times greater than that in healthy soils. The habitat niche breadth of fungal communities was greater in healthy rhizosphere soils than in diseased rhizosphere soils (**Figure 5B**). Moreover, the proportions of both generalists (healthy: 4.30%; diseased: 2.44%) and specialists (healthy: 13.69%; diseased: 12.22%) were higher in healthy rhizosphere fungal communities than in diseased ones (**Figure 5C**). Furthermore, C-score analysis revealed a higher SES in healthy than in diseased rhizosphere fungal communities, confirming enhanced deterministic processes in healthy soils (**Figure 5D**). Collectively, these findings indicate that *R. solanacearum* invasion enhanced stochasticity while weakening determinism in the assembly of tomato rhizosphere fungal communities.

**Figure 5:**
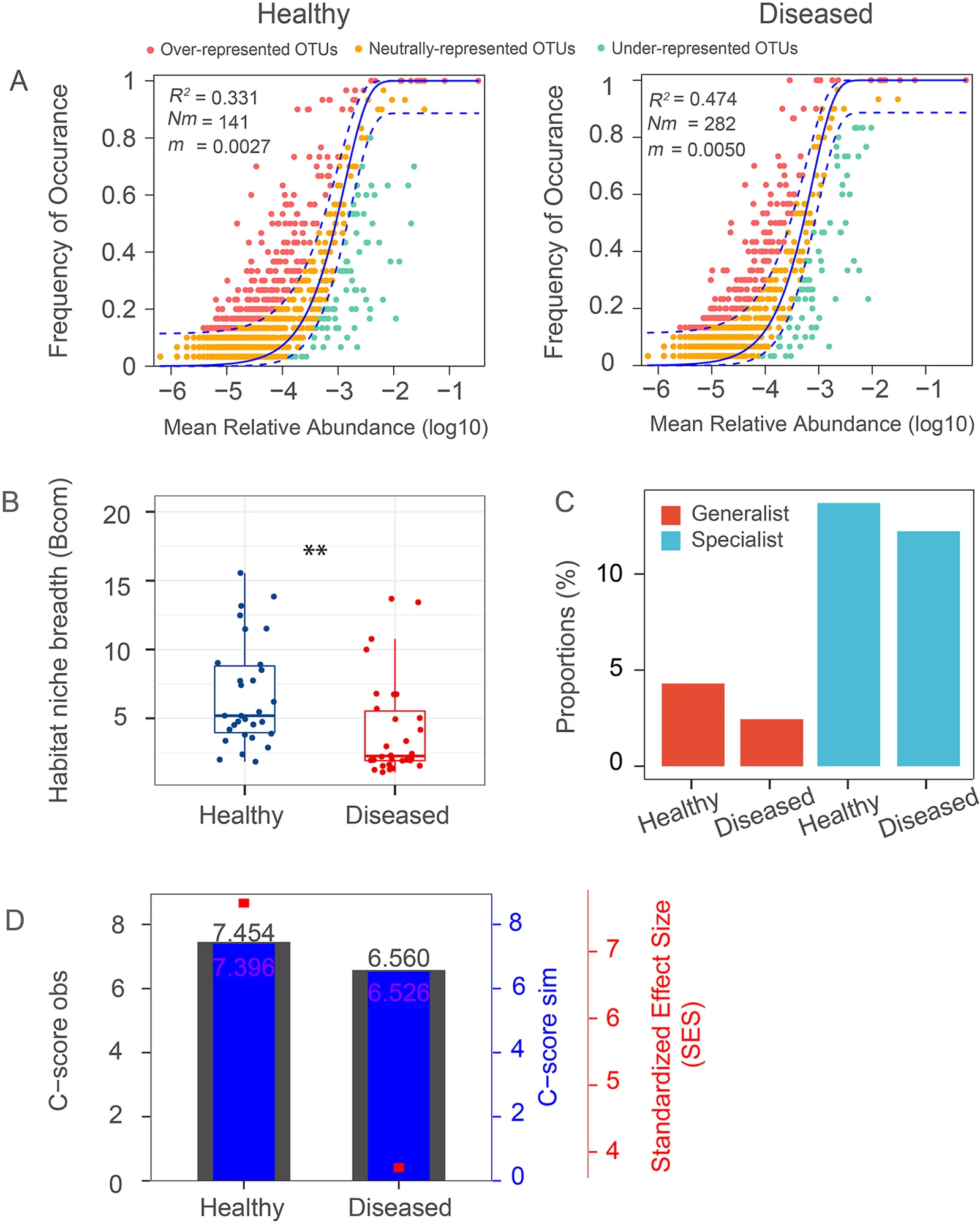
Community assembly processes of fungal communities in healthy and diseased tomato rhizosphere soils. **(A)** Neutral community model (NCM) fitting of fungal communities. The NCM was applied to predict the occurrence frequency of ASVs as a function of their relative abundance. The solid blue and red lines represent the best fit to the neutral model for healthy and diseased soils, respectively; dashed lines indicate the 95% confidence intervals. *R²* values indicate the goodness of fit of the neutral model, *Nm* reflects community dispersal ability, and *m* represents the estimated migration rate. A higher *R²* indicates a greater contribution of stochastic processes to community assembly. **(B)** Habitat niche breadth of fungal communities in healthy and diseased soils. **(C)** Proportions of generalists and specialists in healthy and diseased tomato rhizosphere fungal communities. **(D)** C-score metric based on null models. The observed C-score (C-score_obs_) greater than the simulated C-score (C-score_sim_) indicates non-random co-occurrence patterns. Standardized effect size (SES) < −2 and > 2 indicates aggregation and segregation, respectively. *P < 0.05; **P < 0.01; ***P < 0.001. n = 30.

## 4 Discussion

### 4.1 Soil enzyme activities and fungal community structure in healthy and diseased tomato rhizosphere soils

In support of our first hypothesis, the composition of fungal communities differed markedly between healthy and diseased tomato rhizosphere soils. (**Figure 2B**). The fungal communities were dominated by Ascomycota and Basidiomycota, both of which were more abundant in healthy than in diseased tomato soils (**Figure 2C**). Soil pH was the important factor driving fungal community differentiation between healthy and diseased tomato rhizosphere soils (**Figure 3**). Soil pH strongly influences soil microbial communities across ecosystems (Dong et al., 2026). In addition, the activities of NAG, LAP, and GEMA also significantly shaped the fungal community in healthy tomato rhizosphere soils (**Figure 3**). These environmental factors may have filtered fungal taxa through environmental selection, thereby driving community differentiation (Duan et al., 2025). Soil microorganisms acquire carbon, nitrogen, and phosphorus by secreting extracellular enzymes that hydrolyze complex organic matter into assimilable small molecule nutrients (Nannipieri et al., 2012). Soil nutrients, as substrates for enzymatic reactions, directly constrain microbial metabolic capacity and enzyme activity through their availability and abundance (Stark et al., 2014). In the present study, TN, SOC, and AN contents in healthy tomato rhizosphere soils were markedly higher than in diseased soils (**Table 1**), indicating that healthy rhizosphere soils maintained a more abundant nutrient pool. TN was positively correlated with SOC, a widely documented relationship in terrestrial ecosystem (Manzoni et al., 2010; Chen et al., 2025a). Furthermore, TN and AN were positively correlated with the activities of AG and BG (**Figure 3**), suggesting that increased nitrogen availability may promote microbial investment in carbon-acquiring enzymes (Nannipieri et al., 2012; Chen et al., 2025b). Under sufficient nitrogen supply, microbial enzyme synthesis is no longer constrained by nitrogen limitation, leading to enhanced overall metabolic activity and increased resource allocation to carbon-acquiring enzyme production or other enzymes (Chen et al., 2025b). Increased enzyme production is generally accompanied by enhanced microbial biomass and per-cell metabolic rates (Wortel et al., 2018). The positive correlation between MBC and AG activity partially supported this conclusion (**Figure 3**). NAG hydrolyzes chitobiose to monosaccharides and is a key enzyme for microbial nitrogen acquisition from organic substrates such as fungal cell walls, insect exoskeletons, and crustacean shells (Wieczorek et al., 2014; Gao et al., 2025). Our results showed increased SOC stimulated microbial nitrogen demand, thereby inducing the production of nitrogen-acquiring enzymes to maintain cellular C:N stoichiometric homeostasis (Mooshammer et al., 2014). Collectively, the changes in soil carbon- and nitrogen-related enzyme activities directly reflect microbial investment strategies in carbon and nitrogen resources (Allison et al., 2007). In nutrient rich healthy rhizosphere soils, fungi may tend to increase enzyme activities to accelerate soil nutrient turnover.

A significant negative correlation was observed between pH and PHOS in the study (**Figure 3**), which partially supports earlier reports that decreasing pH enhances phosphorus fixation by Al^3+^ and Fe^3+^ ions, leading to a marked reduction in phosphorus bioavailability (Veloso et al., 2023). In addition, the genera *Aspergillus*, *Penicillium*, *Trichoderma* and *Talaromyces* have been identified as phosphorus-solubilizing microorganisms (Xia et al., 2026; Santoyo et al., 2026; Ribeiro et al., 2026) and were enriched in the healthy tomato rhizosphere soils (**Figure 2D**). The positive correlation between PHOS and these phosphorus-solubilizing microorganisms suggested that healthy tomato soils may promote phosphorus mobilization by enhancing the abundance of these fungi, which secrete organic acids and acid phosphatase to solubilize recalcitrant phosphorus (**Supplementary Table S5**). Among these, *Trichoderma* and *Talaromyces* are widely recognized biocontrol agents (Manathunga et al., 2024), and their enrichment with PHOS activity implies a potential link between phosphorus acquisition and disease suppression (Cao et al., 2024). Some studies indicate that phosphorus, as a necessary plant nutrient, profoundly regulates plant immunologic mechanism, hormone signaling pathways and root exudation, all of which are closely associated with plant disease resistance (Stewart et al., 2001; López-Bucio et al., 2025). For instance, phosphorus stimulates the accumulation of organic acids and flavonoids in plants (Stewart et al., 2001; Huang et al., 2021). However, a recent study showed that the rhizosphere metabolites produced under low phosphorus conditions in cucumber (succinic acid, azelaic acid, threonine, and methionine) recruit beneficial taxa and enhance the resistance of cucumber to Fusarium wilt, whereas the rhizosphere metabolites produced under high phosphorus conditions promote the growth of pathogens (Liu et al., 2026). Thus, the interactions among phosphorus, microorganisms, and plant disease resistance are highly complex, influenced by pathogen type, plant genotype, and rhizosphere microbial conditions (Cao et al., 2024; Huang et al., 2024). Our findings suggest soil phosphorus and acid phosphatase as potential indicators of plant disease resistance.

### 4.2 Fungal co-occurrence networks in healthy and diseased tomato rhizosphere soils

Network analysis provides valuable insights into microbial interactions in soil-borne disease research (Chen et al., 2024; Han et al., 2025). In the study, Healthy tomato rhizosphere fungal networks had more nodes, more edges, and higher natural connectivity than diseased networks (**Figure 4**), indicating greater complexity and stability of the healthy networks, which partially supports our first hypothesis. This difference may be attributed to three factors. First, the complexity of soil microbial networks increases with microbial diversity (Wu et al., 2024). Our results showed higher fungal diversity in healthy rhizosphere soils (**Figure 2A**), which contributes to increased potential interactions. Second, the abundances of both generalists and specialists were higher in healthy than in diseased soils (**Figure 5C**). Generalists, with broader niche breadth, are distributed across multiple network modules and primarily maintain overall network connectivity; specialists, with higher niche specialization, form dense connections within modules and mainly enhance functional redundancy (Clavel et al., 2011; Xu et al., 2022; Liu et al., 2023). The enrichment of both generalists and specialists in healthy soils may enhance network connectivity and connections within modules, collectively strengthening network complexity and disease resistance. In contrast, the reduction of both groups in diseased soils may lead to decreased network connectivity and functional redundancy, thereby compromising the ability of microbial networks to resist pathogen invasion. The connectors of microbial networks are typically occupied by generalist taxa with broad niche breadth, as they can persist across different microhabitats and resource patches (Hao et al., 2025). In the study, the connector proportion was greater in healthy than in diseased tomato rhizosphere fungal networks (**Figure 4D**; **Supplementary Tables S3, S4**), aligning with the higher generalist abundance observed in healthy soils. Notably, the fungal taxa that differed between healthy and diseased tomato rhizosphere soils in this study played important topological roles in the co-occurrence networks. For example, *Penicillium* and *Aspergillus* were not only significantly enriched in healthy soils as differential taxa, but also their respective ASVs, ASV_9 and ASV_26, served as key connectors in the co-occurrence networks (**Figure 2D**; **Supplementary Tables S3, S4**). Third, broader community-level niche breadth is expected to promote species coexistence and thereby enhance network complexity (Jiao et al., 2020; Liu et al., 2023). In our study, the habitat niche breadth of fungal communities at the community level was higher in healthy than in diseased tomato rhizosphere soils (**Figure 5B**). The highly connected fungal co-occurrence networks indicate that healthy rhizosphere soils may offer more shared niches, facilitating greater coexistence of fungal communities (Jiao et al., 2020).

### 4.3 The assembly mechanisms of fungal microbial communities in healthy and diseased tomato rhizosphere soils

In the present study, deterministic processes governed fungal community assembly in healthy tomato rhizosphere soils, whereas stochastic processes dominated in diseased soils (**Figure 5**). There were two possible reasons. First, pathogen invasion alters the composition and quantity of root exudates, weakening the host’s capacity to recruit beneficial microbial taxa (He et al., 2026). In a study of *Verticillium* wilt in *Astragalus*, invasion by *Verticillium dahliae* shifted rhizosphere microbial community assembly from determinism to stochasticity, accompanied by a decline in α-diversity due to the proliferation of opportunistic pathogens and the reduction of beneficial taxa (Ai et al., 2026). The deterministic recruitment of beneficial microorganisms is considered essential for mitigating environmental stress (Manathunga et al., 2024). In line with this, the relative abundances of genera *Trichoderma*, *Talaromyces*, *Mortierella*, and *Penicillium* were lower in diseased than in healthy rhizosphere soils (**Figure 2D**). Second, root cell damage caused by pathogen infection also releases large quantities of low-molecular-weight organic carbon, temporarily relieving substrate limitation and thereby increasing the reliance of microbial growth on stochastic processes, including dispersal and drift (Ai et al., 2026; Adeniji et al., 2026). This is supported by the higher dispersal rate of fungal communities observed in diseased rhizosphere soils compared to healthy soils in the study (**Figure 5A**). These two mechanisms independently weaken deterministic selection or enhance stochastic processes, jointly contributing to the stochastic-dominated assembly pattern in diseased soils. Notably, some studies have reported that stochastic processes dominate rhizosphere community assembly in healthy plants (Wen et al., 2022), a discrepancy that may be attributed to differences in spatial scale, nutrient availability, resource competition, and host characteristics (Shi et al., 2018; Gao et al., 2020; Wang et al., 2024).

Collectively, deterministic processes in healthy tomato rhizosphere fungal communities may reflect strong host-mediated selective pressure exerted through metabolic activities and immune surveillance, which drives the formation of non-random interspecific interactions and manifests as increased network complexity (He et al., 2026; Ai et al., 2026). The efficient operation of host deterministic selection in healthy rhizosphere soil benefits from a relatively high community level niche width, which provides sufficient ecological niches for different functional groups (Jiao et al., 2020; Liu et al., 2023). In diseased rhizosphere fungal communities, increased stochasticity may reflect weakened host selection, leading to a decline in beneficial functional groups (Shi et al., 2025). Under such conditions, species coexistence becomes increasingly reliant on dispersal and ecological drift, ultimately leading to network simplification and randomization. In support of our second hypothesis, these findings indicate that fungal community assembly processes play a critical role in shaping co-occurrence network structure.

## 5 Conclusions

In this study, we compared the community structure, co-occurrence networks, and assembly mechanisms of rhizosphere fungal communities between healthy and *R. solanacearum*-infected tomato plants. Soil pH and PHOS activity drove fungal community differentiation between healthy and diseased soils, and their negative correlation suggests a potential link between phosphorus mobilization and disease suppression. Beneficial genera including *Penicillium*, *Aspergillus*, *Trichoderma*, and *Talaromyces* were enriched in healthy rhizosphere soils. Following *R. solanacearum* invasion, stochastic processes in fungal community assembly were generally enhanced, while deterministic processes were weakened. These findings indicate that pathogen invasion may shift rhizosphere fungal community assembly from determinism toward stochasticity, accompanied by reduced proportions of generalists and specialists, as well as network simplification and decreased stability. This study provides important evidence for understanding the formation mechanisms of rhizosphere fungal communities following pathogen invasion and the microbiome-mediated suppression of soil-borne diseases. The invasion of *R. solanacearum* inevitably leads to direct antagonism or resource competition with resident microbial communities. Future studies are needed to further investigate whether the changes in fungal communities are related to cross-kingdom interactions between fungi and bacteria.

## Data Availability

Raw sequencing data have been deposited in the NCBI Sequence Read Archive (BioProject accession: PRJNA1463964).
